# Supplementary material for: Low cost exoskeleton manipulator using bidirectional triboelectric sensors enhanced multiple degree of freedom sensory system
Source: Nat Commun. 2021 May 11;12:2692. doi: 10.1038/s41467-021-23020-3 (PMC8113469; doi:10.1038/s41467-021-23020-3)
Supplement: Supplementary file 1 — Supplementary information [file 41467_2021_23020_MOESM1_ESM.pdf]

Supplementary Information for

Low cost exoskeleton manipulator using bidirectional triboelectric sensors enhanced multiple degree of freedom sensory system

Minglu Zhu, Zhongda Sun, Tao Chen,* and Chengkuo Lee*

*Corresponding author: elelc@nus.edu.sg (C. L.); chent@suda.edu.cn (T.C.)

Supplementary Note 1. Optimization strategy for high resolution and high rotation speed sensing
Supplementary Note 2. Response latency for the switching of the direction
Supplementary Note 3. Customized print circuit board (PCB) for optimizing multichannel sensing of triboelectric output.
Supplementary Note 4. Kinetic analysis of punching force estimation via TBD sensors
Supplementary Note 5. Optimization strategy of increasing degree of freedoms of shoulder module
Supplementary Fig. 1. Photos of the as-fabricated sensors and the assembled exoskeleton arms
Supplementary Fig. 2. FEM simulation of surface electrical potential between the copper spring and the PTFE gratings
Supplementary Fig. 3. Optimization strategy for high resolution and high rotation speed sensing
Supplementary Fig. 4. Experiment for the robustness of long-term usage
Supplementary Fig. 5. Comparison of the output signals under varied environmental conditions
Supplementary Fig. 6. Evaluation of response latency during the switching of the direction for different designs of switches
Supplementary Fig. 7. Signal processing circuit and transmission for the multichannel sensors
Supplementary Fig. 8. Kinetic analysis of punching force estimation via TBD sensors
Supplementary Fig. 9. Optimization strategy of shoulder module for three DOFs
Supplementary Table 1. Relationship among the measured angles of α, β, and γ along the punching path

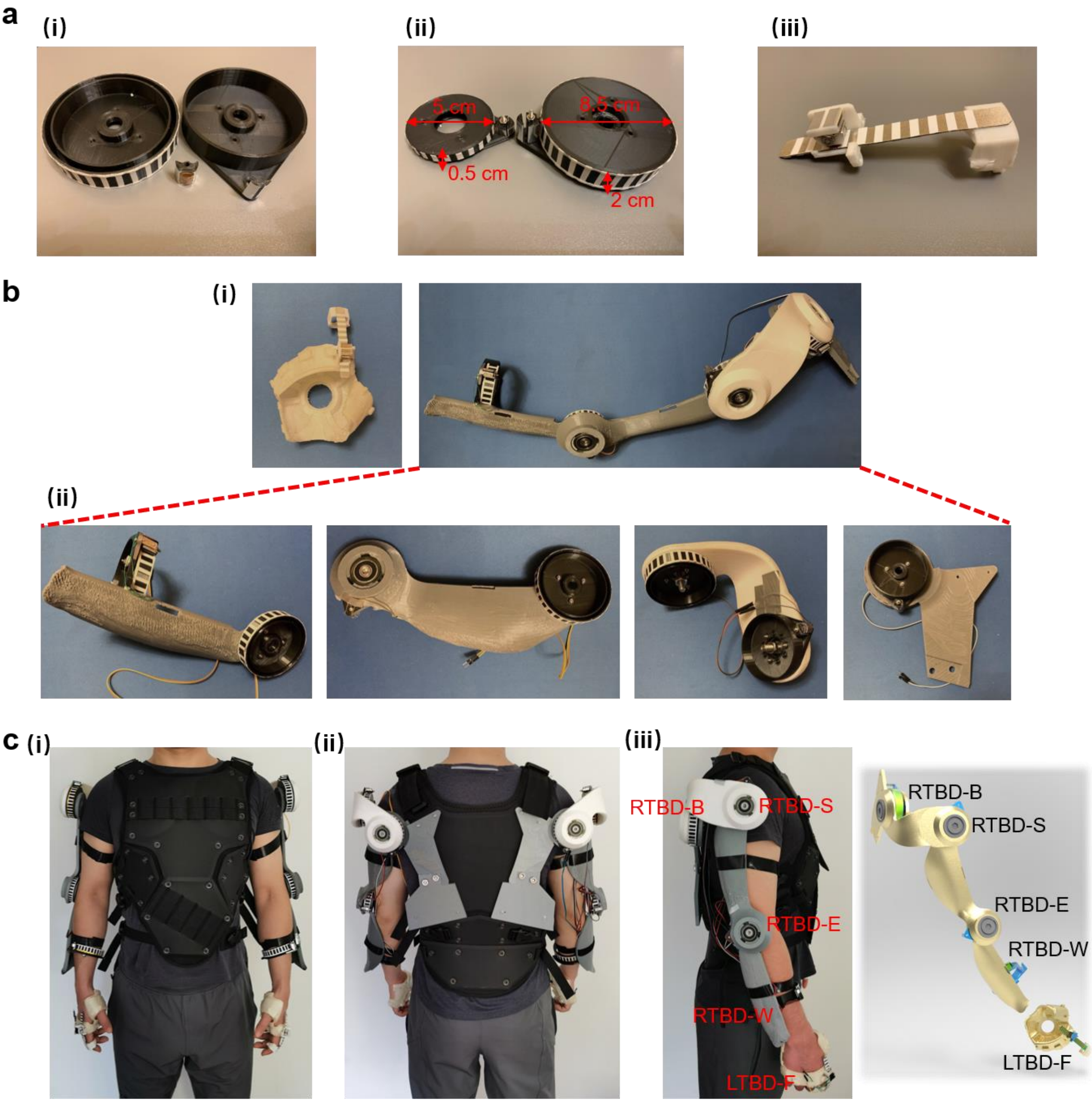

Supplementary Fig. 1. Photos of the as-fabricated sensors and the assembled exoskeleton arms. **a** (i) Main components of triboelectric bi-directional (TBD) sensor, (ii) assembled TBD sensors, including two different sizes, and (iii) assembled linear TBD sensor. **b** (i) Integration of the sensors on the exoskeleton arm and glove, and (ii) major components of the exoskeleton arm, including forearm, upper arm, L-shaped shoulder module, back support (from left to right). **c** Exoskeleton after wearing (i) front view, (ii) back view, and (iii) side view and schematics of exoskeleton with the rotational triboelectric bi-directional back (RTBD-B) sensor, the rotational triboelectric bi-directional shoulder (RTBD-S) sensor, the rotational triboelectric bi-directional elbow (RTBD-E) sensor, the rotational triboelectric bi-directional wrist (RTBD-W) sensor, and the linear triboelectric bi-directional finger (LTBD-F) sensor. Photo credit: Minglu Zhu, National University of Singapore.

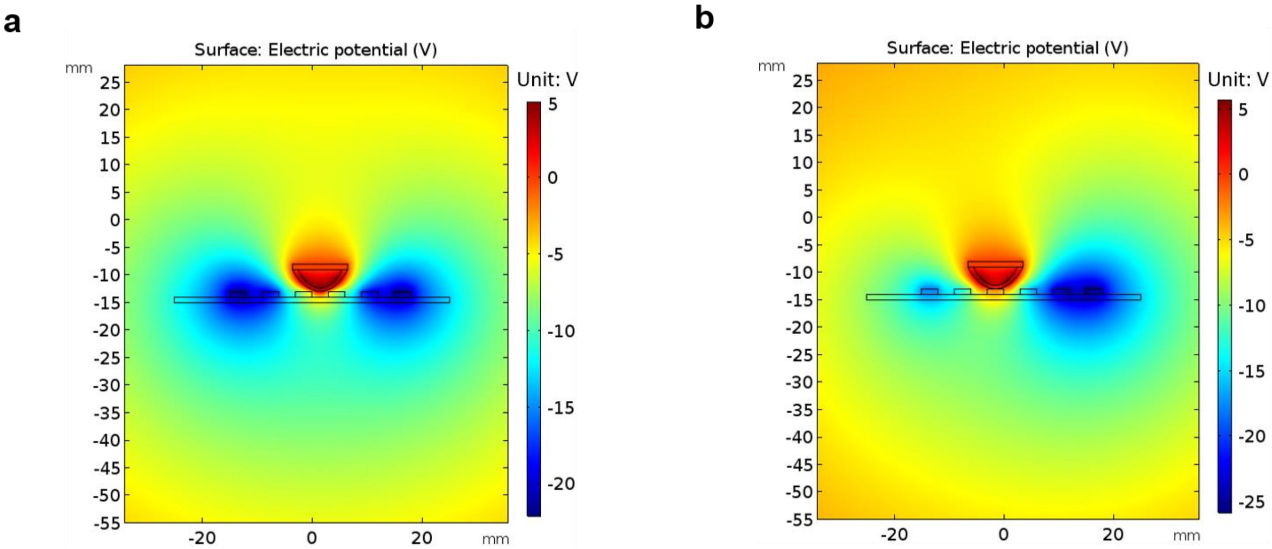

Supplementary Fig. 2. FEM simulation of surface electrical potential between the copper spring and the PTFE gratings a separation and **b** contact states.

Supplementary Note 1. Optimization strategy for high resolution and high rotation speed sensing

As mention in the main manuscript, the small spacing of the grating pattern experiences the severe loss of output peaks at high rotation speed, i.e., 300 revolutions per minute (RPM), as shown in Supplementary Fig. 3a. Compare to the signals at 200 RPM, this issue of the resolution loss will affect the signal processing for manipulation purpose, and cause the inconsistency between the actual human motion and the controlled objects. The main reason of this problem is the width of the copper spring which is used to slide across the PTFE gratings (Supplementary Fig. 3b(i)). As the spacing keep decreasing, there will be no enough space for the copper spring to achieve a full separation with the previous grating, as illustrated in Supplementary Fig. 3b(ii). In another word, the copper spring will connect both of the adjacent gratings, which will affect the charge transfer due to the incomplete contact-separation cycle. Hence, to solve this issue, an effective approach is to reduce the width of the copper spring, in order to re-establish the complete contact-separation cycles.

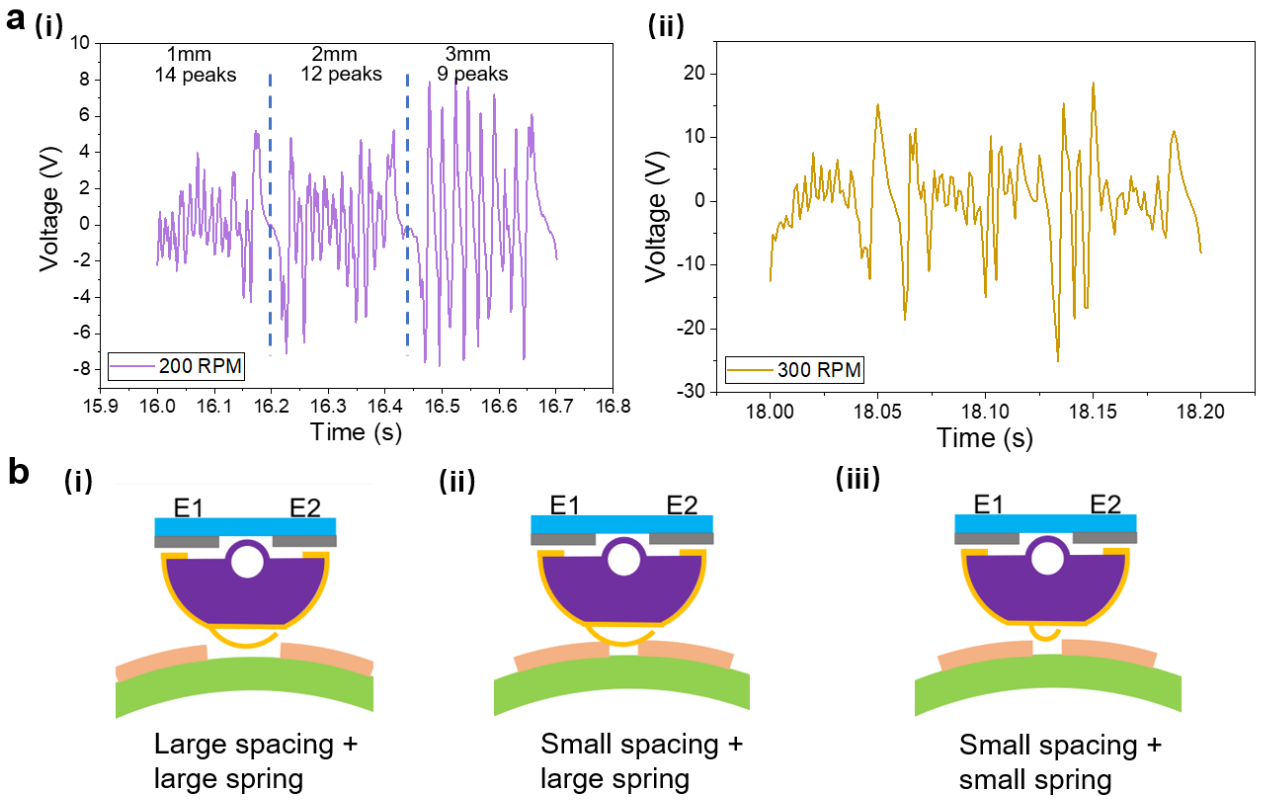

Supplementary Fig. 3. Optimization strategy for high resolution and high rotation speed sensing. **a** Comparison of the output signals from the characterization of the varied spacing, (i) the signals for 200 revolutions per minute (RPM) rotation, and (ii) the signals from 300 RPM rotation. **b** Schematics of the sensor designs of (i) large spacing with large spring, (ii) small spacing with large spring, and (iii) small spacing with small spring.

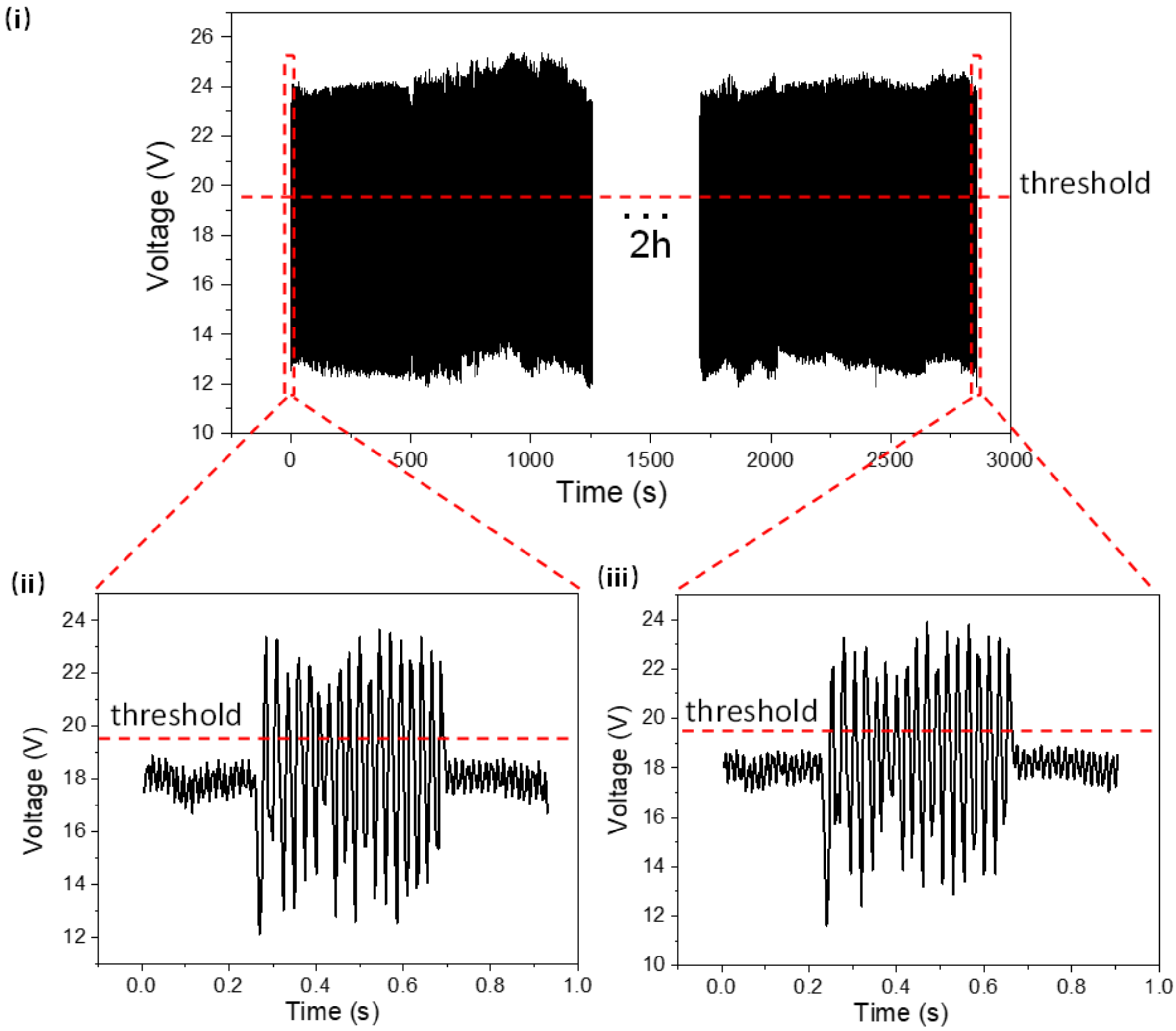

Supplementary Fig. 4. Reliability experiment for the signal stability of long-term usage. (i) Reliability test of the output signals for long-term operation of 3 hours, the red dashed lines are the tunable threshold voltage from the comparator circuit. (ii) Output signals recorded at the beginning of the test. (ii) Output signals recorded at the end of the test.

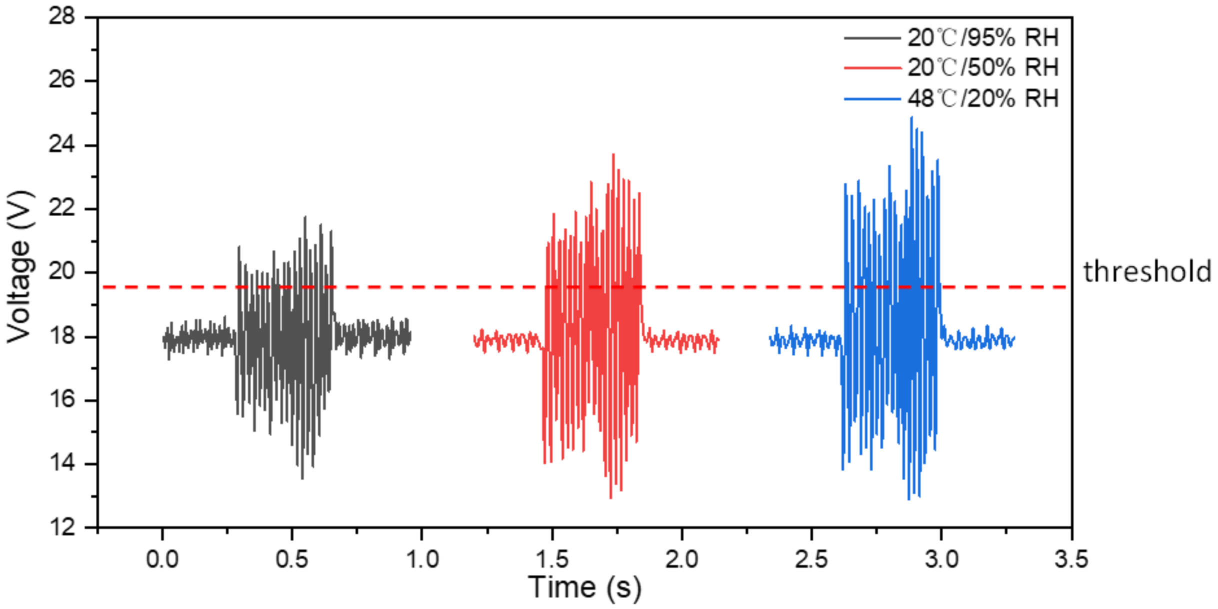

Supplementary Fig. 5. Comparison of the output signals under varied environmental conditions. (i) 20℃ and 95% relative humidity, (ii) 20℃ and 50 % relative humidity, (iii) 48℃ and 20% relative humidity. Rotation speed: 100 revolutions per minute (RPM).

Supplementary Note 2. Response latency for the switching of the direction

As a bi-directional sensor with a pendulum-like switching mechanism, the design of the switch structure may affect the response latency of the sensor, and eventually determine the state of synchronization between the human motion and the controlling objects. To investigate this issue, two designs of switches were proposed. The switch with shaper corner has the gaps of 1.5 mm at neutral state, where as the switch with flatter angle has 3 mm gaps. In order to generate the triboelectric output for monitoring the switching process, the PTFE as negative triboelectric materials were attached on E1 and E2, and the aluminum film on switch was positive triboelectric materials. Both of two switches were tested under two rotation speed, 10 RPM and 100 RPM. As shown in Supplementary Fig. 6, the red negative peak represents the separation of E1, and the black positive peak indicates the contact of E2. Based on the enlarged view of the peaks of the separation and the contact during switching, the response latency is about a few tens of milliseconds, which can be considered as small for the practical applications. These gaps can also be further reduced by modifying the design of the switch.

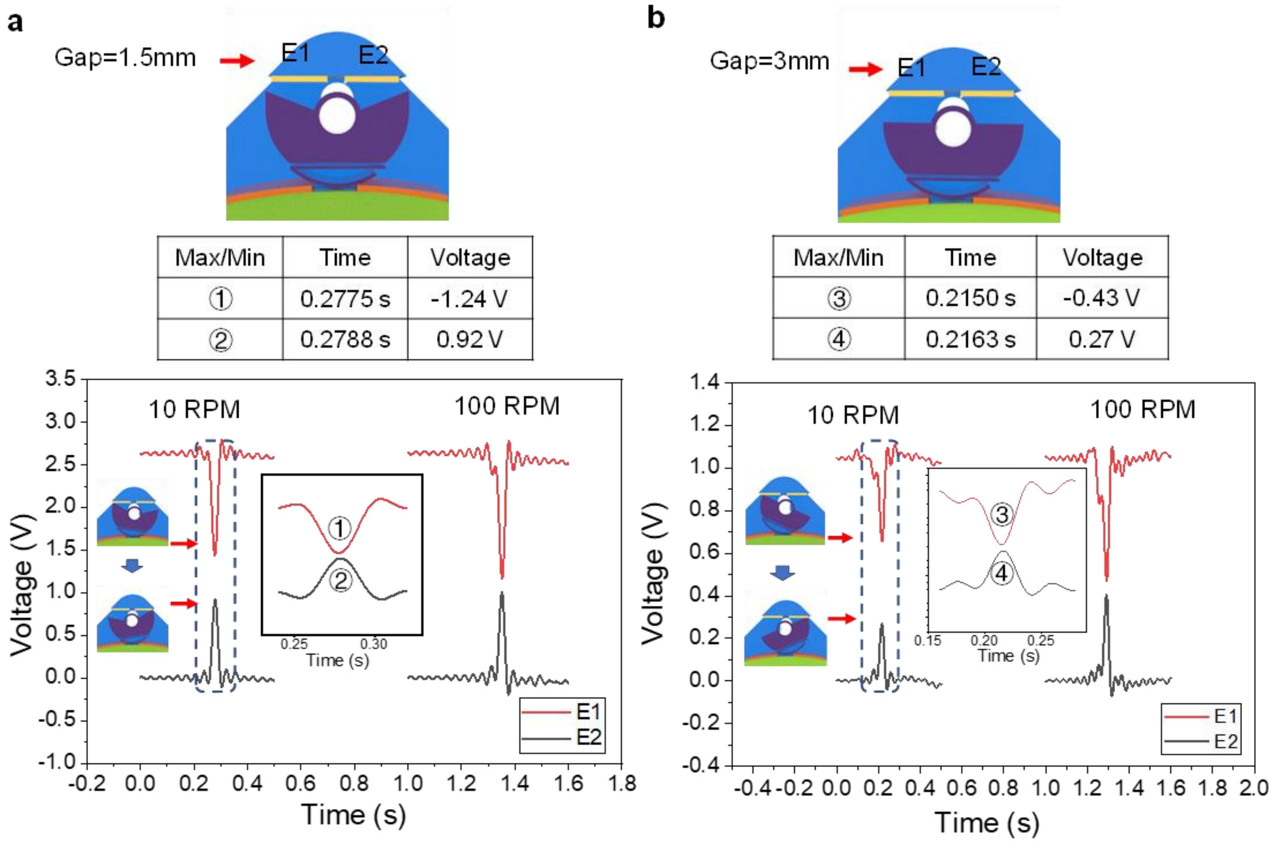

Supplementary Fig. 6. Evaluation of response latency during the switching of the direction for different designs of switches. a Switch with 1.5 mm gap at neutral state, and the corresponding signals from E1 and E2 electrodes during the switching, the red negative peak represents the separation of E1, and the black positive peak indicates the contact of E2, the enlarged graph of the dashed box, as well as the time and the peak voltages of the separation (1) and contact (2) signals are provided. **b** Switch with 1.5 mm gap at neutral state, and the corresponding signals from E1 and E2 electrodes during the switching, the enlarged graph of the dashed box as well as the time and the peak voltages of the separation (3) and contact (4) signals are provided.

Supplementary Note 3. Customized print circuit board (PCB) for optimizing multichannel sensing of triboelectric output

The direct triboelectric signal readout from the commercialized microprocessor with a single analog-to-digital converter (ADC), such as Arduino, will engage with the cross-talk and the large noise issues. As shown in Supplementary Fig. 7a, the conditioner unit consists of operational amplifier and low pass filter is applied to solve those problems, while maintaining the floating signal to acquire the entire triboelectric waveform in Supplementary Fig. 7b(i). Furthermore, the unified output peaks are preferred to ensure the convenient programming of the peak detection. Hence, the comparator circuit is also introduced to convert the original pulse waveform into the square waveform (Supplementary Fig. 7b(ii)). Next, by utilizing the edge inspection code to detect those square peaks, the rotation signals can be expressed into the desired command, i.e., 1 for one peak from clockwise rotation, 2 for one

peak from counter-clockwise rotation. With the TCP/IP serial communication between Python and Unity, the virtual character can then be controlled in real-time.

For robotic manipulation, the command with angular information need to be converted into hexadecimal format before send to the servo controller. A communication cable for USB to TTL is used for data transmission.

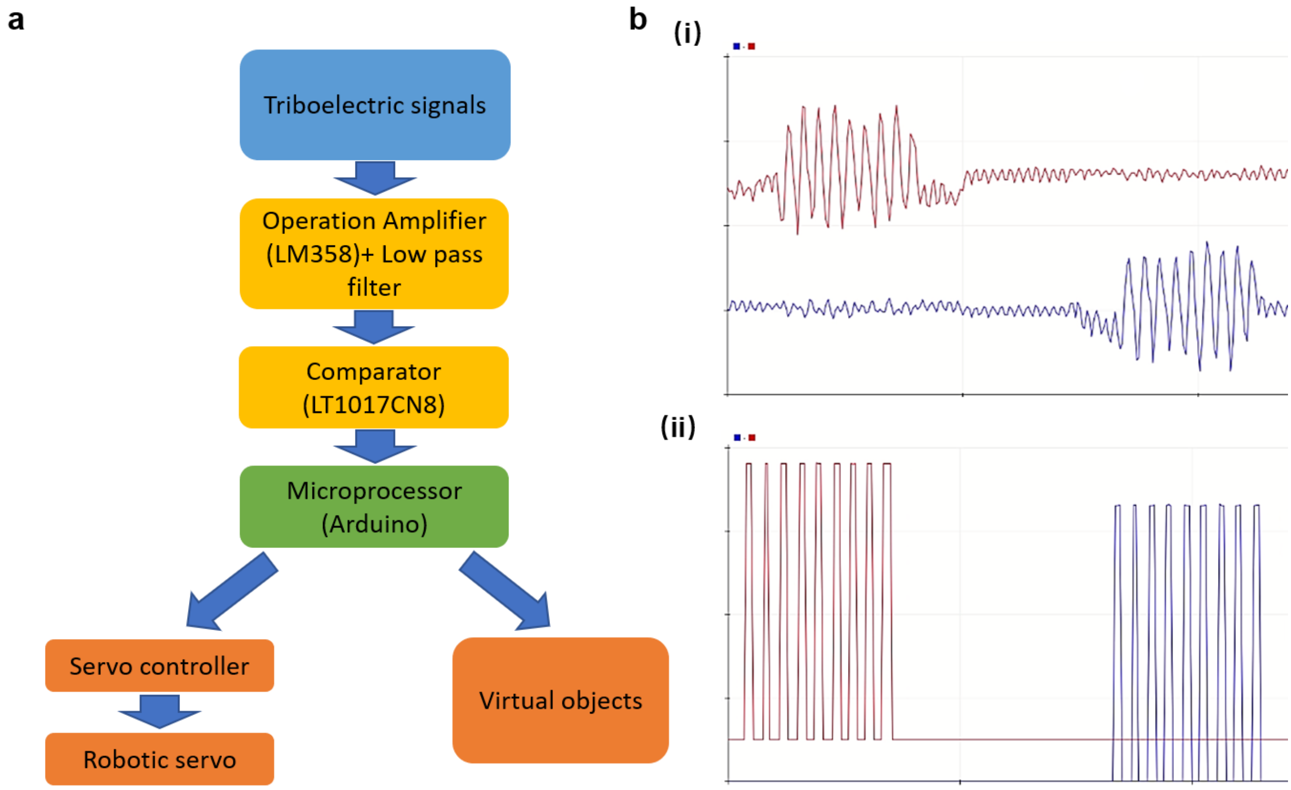

Supplementary Fig. 7. Signal processing circuit and transmission for the multichannel sensors. **a** Flow chart of signal processing and transmission. **b** (i) triboelectric signals before the comparator, and (ii) after comparator.

Supplementary Note 4. Kinetic analysis of punching force estimation via rotational triboelectric bi-directional (RTBD) sensors

The straight punching is selected for a simplified analysis of the boxing dynamics. In this case, the shoulder joint is considered as a fixed point by assuming no torso motion. The path of punching is a direct forward punching, and hence, the factor of eccentricity c equals to 0 since all of center of three joints (shoulder, elbow, and fist) are aligned in a horizontal line. In general, this model can then be treated as a slider crank mechanism for further investigation. As illustrated in Supplementary Fig. 8a and 8b, the length between the shoulder and the fist can be expressed as:

$$L = L_1 \cos\alpha + L_2 \cos\beta \qquad (1)$$

Whereas L is the length between the shoulder and the fist, L_1 is the length of the upper arm, L_2 is the length of the forearm, α is the angle between the upper arm and the center line of punching, β is the angle between forearm and the center line of punching.

As the largest distance the shoulder and the fist is:

$$L_{max} = L_1 + L_2 \quad (2)$$

Hence, the displacement of the fist can be calculated as:

$$d = L = (L_1 \cos\alpha + L_2 \cos\beta) \qquad (3)$$

Let $\lambda = L_1/L_2$

$$d = L_1 \cos\alpha + L_2\left(\sqrt{1-(\sin\alpha)^2\lambda^2}\right) \qquad (4)$$

By utilizing the function of rotation speed sensing of the proposed sensors, the angular speed can be detected as ω_S, ω_E, for the rotation of shoulder and elbow, respectively. Meanwhile, the velocity of the fist can be determined as:

$$v = \frac{dd}{dt} = \frac{dd}{d\alpha}\cdot\frac{d\alpha}{dt} \qquad (5)$$

Since $\omega_S = \frac{d\alpha}{dt}$, the velocity of the fist can then be calculated as:

$$v = -L_1\omega_S \sin\alpha - L_2\omega_S \frac{\lambda^2 \sin\alpha\cos\alpha}{\sqrt{1-\lambda^2\,(\sin\alpha)^2}} \qquad (6)$$

Furthermore, the acceleration of the fist can be calculated as:

$$a = \frac{dv}{dt} = \frac{dv}{d\alpha}\cdot\frac{d\alpha}{dt} \qquad (7)$$

Next, based on conservation of momentum, two equations (Eq. 4 and Eq. 5) are applied to define the forces exerted from the fist and received by the target, respectively.

$$F_f = ma \qquad (8)$$

$$mv - mv_0 = F_t t \qquad (9)$$

Where m is the mass of the whole arm, a is the acceleration of the fist, v is the final velocity when contacting with target, v_0 is the initial velocity at the beginning, F_f is the forces generated from the fist, F_t is the force received by the target, t is the time period from the contacting of the target to the complete stop.

To verify the proposed kinetic analysis for the estimation of the punching force, a force meter is applied to determine the errors. As shown in Supplementary Fig. 8a(iii), the punching forces of 4 kg and 6 kg were measured for the linear fist velocity of 1 m/s and 1.5 m/s, respectively. By assuming the contact time period as 0.1 s, the calculated force are 40 N and 90 N, respectively. According to Fig. 6g(ii), the estimated forces (50 N and 87 N) via the data of the rotation TBD sensor can be considered as reasonable, with the errors of 25% and 3%.

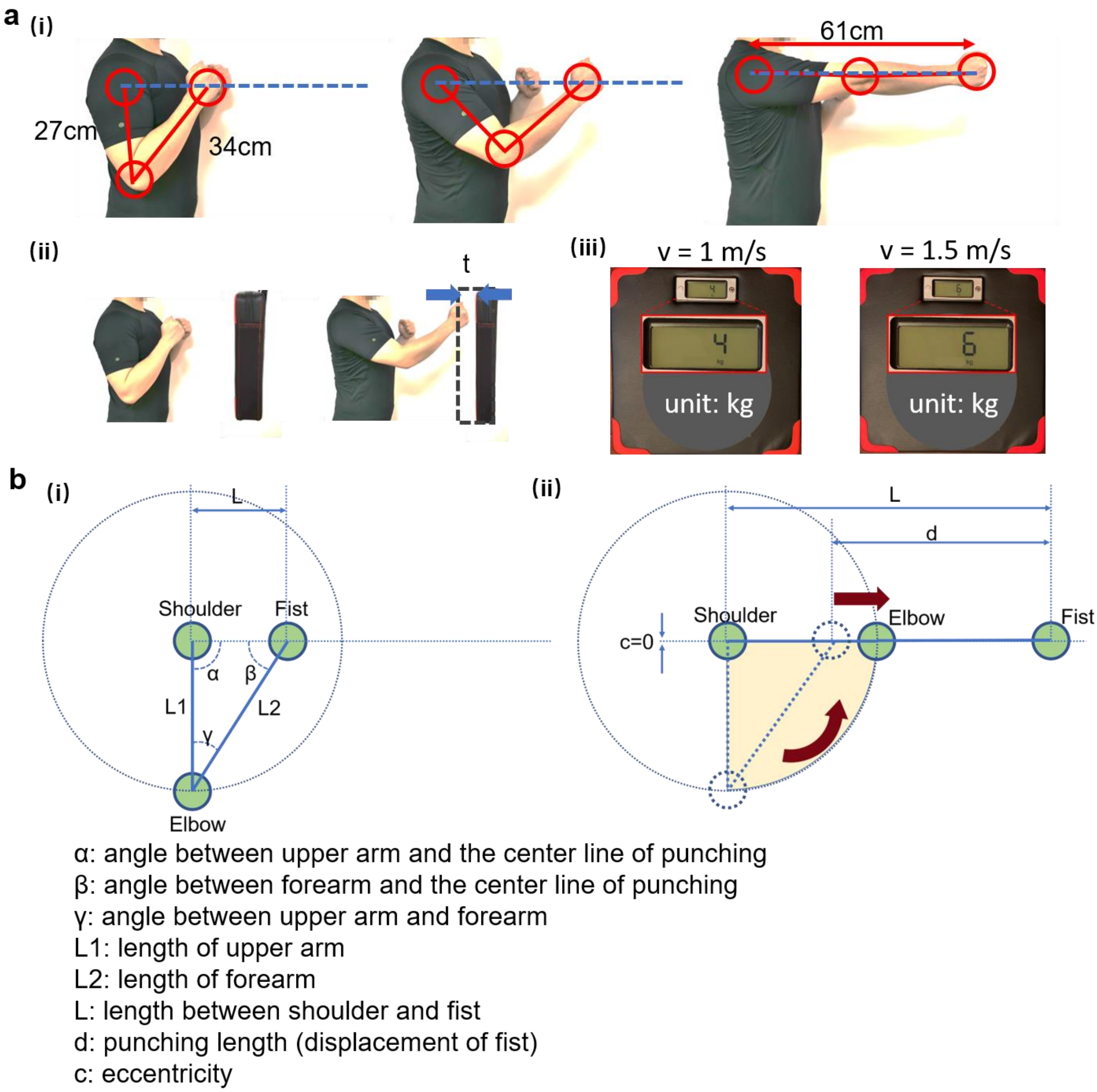

Supplementary Fig. 8. Kinetic analysis of punching force estimation via rotational triboelectric bi-directional (RTBD) sensors. a (i) Photos and schematics of straight punch motion. (ii) Photos and schematics of stopping the punch by target. (iii) Measured punching force at the speed of 1 m/s and 1.5 m/s. **b** Schematics of kinetic analysis: (i) initial state, and (ii) final state.

Supplementary Table 1. Relationship among the measured angles of α, β, and γ along the punching path.

Position of fist (cm)	α (shoulder) (degree)	β (fist) (degree)	γ (elbow)(degree)
18	90	60	30
23	83	57	40
27	77	53	50
32	70	50	60
36	63	47	70
40	56	44	80
44	49	41	90
48	42	38	100
51	36	34	110
53	31	29	120
55	27	23	130
57	23	17	140
59	17	13	150
60	10	10	160
60.5	4	6	170

Supplementary Note 5. Optimization strategy of increasing degree of freedoms (DOFs) of shoulder module

As shown in Supplementary Fig. 9a, except two DOFs demonstrated in the main manuscript, the human shoulder actually possesses the additional DOF, which is known as flexion/extension motion shown in Supplementary Fig. 9a(iii) (marked as flexion/extension 2). To provide the sensing capability of this DOF, the current shoulder module can be further optimized by dividing it into two separate parts, and reassembled with a hinge. A small size RTBD sensor can be mounted on the hinge and in charge of the detection of the flexion/extension 2. This modified version of shoulder module is able to provide better projection of the actual human motions with three DOFs. The current design with two DOFs can still achieve the manipulation functions in 3D space.

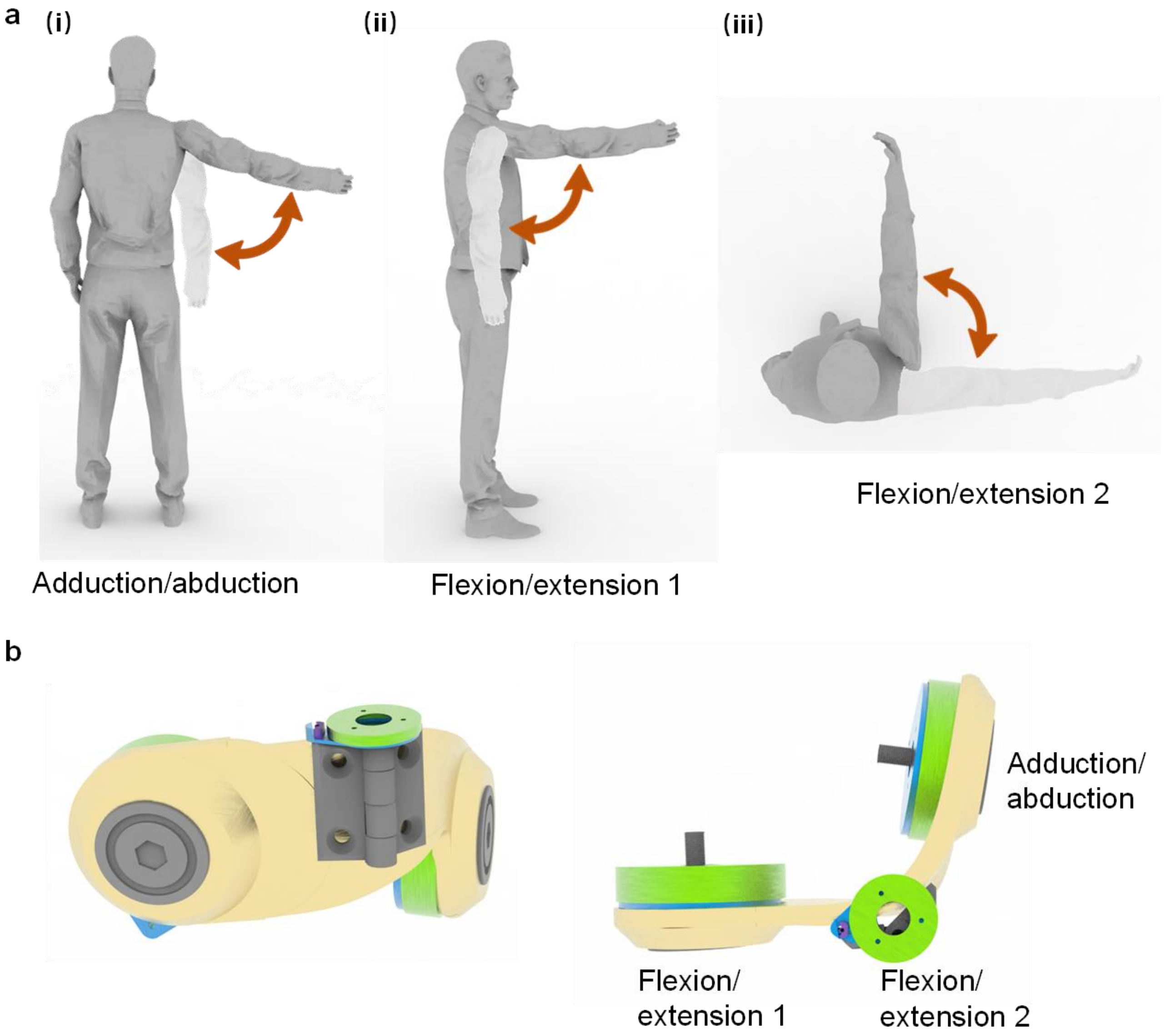

Supplementary Fig. 9. Optimization strategy of shoulder module for three degree of freedoms (DOFs). a Three DOFs of shoulder motions. **b** Optimized design of shoulder module for realizing the monitoring of three DOFs.
